# Dermoscopic Features of Non-Infectious Balanoposthitis

**DOI:** 10.3390/jcm14238438

**Published:** 2025-11-28

**Authors:** Aleksejs Zavorins, Kristīne Nevidovska, Jūlija Voicehovska

**Affiliations:** 1 Department of Dermatology and Venereology, Riga Stradins University, LV-1007 Riga, Latvia; 2 Pathology Center, Riga East Clinical University Hospital, LV-1038 Riga, Latvia; 3 Department of Internal Diseases, Riga Stradins University, LV-1007 Riga, Latvia; julija.voicehovska@rsu.lv

**Keywords:** dermoscopy, balanoposthitis, psoriasis, lichen planus, lichen sclerosus, Zoon’s balanoposthitis

## Abstract

**Background/Objectives**: Balanoposthitis encompasses a variety of non-infectious conditions that, in some cases, are risk factors for penile cancer. Clinical signs of non-infectious balanoposthitis are often unspecific, whereas dermoscopic features of dermatoses in the genital area can be altered. Current knowledge of the dermoscopy of balanoposthitis is incomplete. This study aimed to evaluate the dermoscopic features of non-infectious balanoposthitis and correlate these features with distinct balanoposthitis types. **Methods**: Patients with histopathologically confirmed non-infectious balanoposthitis (N = 78) were enrolled in a prospective study, including lichen planus, psoriatic, lichen sclerosus and Zoon’s balanoposthitis. Dermoscopic features were compared between each type of non-infectious balanoposthitis using non-parametric statistics. **Results**: White and purple reticular lines and purple focal structureless areas were dermoscopic variations in Wickham striae were associated with lichen planus, as well as curved linear vessels at the periphery of Wickham striae. Dotted and linear curved vessels in an unspecific arrangement were associated with lichen sclerosus. Other structures included white lines in an unspecific arrangement, white dots and globules and structureless areas. Red and purple globules in lichen sclerosus represented purpura. Notable dermoscopic features that were associated with psoriatic balanoposthitis were yellow-white globules that represented pustules, as well as dotted vessels in a uniform arrangement and patchy white scales. **Conclusions**: Distinct types of non-infectious balanposthitis are associated with certain dermatoscopic features. Wickham striae have a variable dermoscopic presentation. Some dermoscopic structures can mimic Wickham striae, but vascular patterns can help in differentiating these cases. Pustules can be observed dermoscopically in some psoriasis cases.

## 1. Introduction

Balanoposthitis is the inflammation of the glans penis and the foreskin. A common misconception among clinicians is to presume that balanoposthitis has an infectious cause, particularly fungal. Contrary to popular belief, a large portion of balanoposthitis cases (22.64–46.1%) are of non-infectious etiology, including lichen planus (LP), lichen sclerosus (LS), psoriatic (PBP) and Zoon’s balanoposthitis (ZBP) [1,2]. Although some specific signs, such as Wickham striae (WS) in the case of LP or extragenital lesions in the case of psoriasis, can help in diagnosing the condition, in many cases balanoposthitis can be an isolated finding, with an unspecific clinical presentation such as erythema and erosions on the glans or the foreskin [1,3].

Balanoposthitis has been implicated as a risk factor for penile cancer. In particular, LP is a risk factor for the development of penile intraepithelial neoplasia (PIN)—a precursor lesion of an invasive squamous cell carcinoma (SCC)—while LS is considered a direct risk factor for invasive penile SCC [4]. Therefore, a prompt differentiation of balanoposthitis types is crucial for the optimal management of the condition. Dermoscopy is a non-invasive examination method that is no longer reserved for diagnosing neoplastic conditions. Dermoscopic features of inflammatory cutaneous disease have been widely described. Application of dermoscopy can potentially aid in diagnosing non-infectious balanoposthitis and avoiding unnecessary biopsies, monitoring the response to treatment of the condition or its progression, as well as pinpointing the area that should be biopsied to rule out malignant transformation. Dermoscopic patterns of LP and psoriasis lesions have been reported to differ according to the involved body area [5,6]. The mucocutaneous surface of the glans penis can be exposed to chemical irritation and maceration due to occlusion, as well as friction due to sexual practices. These factors can alter clinical and dermoscopic features of common dermatoses in the genital area [7]. Currently, there is a limited number of small-scale studies that focus on dermoscopic features of balanoposthitis [8,9,10]. The use of unstandardized and metaphorical terminology complicates the data analysis and its reproducibility [11,12]. The objective of our study was to evaluate the dermoscopic features of non-infectious balanoposthitis using primarily descriptive, reproducible terminology and to correlate these features with distinct types of non-infectious balanoposthitis (NIBP).

## 2. Materials and Methods

Seventy-three patients who attended our outpatient clinic from November 2018 to January 2024 with NIBP were enrolled in this prospective study. Inclusion criteria were chronic recurrent course of balanoposthitis for at least 4 weeks; age ≥ 18 years; histopathologically confirmed diagnosis. Exclusion criteria were (a) circumcision; (b) presence of sexually transmitted diseases or infectious balanoposthitis; (c) use of topical or systemic therapy to treat balanoposthitis during the previous 4 weeks; (d) neoplastic disease of the glans or the prepuce; (e) ambiguous histopathology report (e.g., descriptive histopathological report with no specific diagnosis indicated, lack of clinicopathological correlation).

Dermoscopic images of the lesions on the penile glans were acquired at 20× magnification (Medicam 1000s, FotoFinder Systems GmbH, Bad Birnbach, Germany). A 3 mm punch biopsy was performed afterwards in a dermoscopically preselected area to rule out malignancy and verify the diagnosis. Patients were grouped according to the type of NIBP, namely, lichen planus (LPBP), psoriatic (PBP), lichen sclerosus (LSBP), Zoon’s (ZBP) and non-specific (NSBP) balanoposthitis. The histopathological report of patients diagnosed with NSBP contained epidermal spongiosis and perivascular inflammatory infiltrate in the upper dermis. Due to the lack of standardized dermoscopic terminology for inflammatory genital conditions, the selections of dermoscopic features were partly based on the terminology for non-neoplastic dermatoses of the International Dermoscopy Society (IDS). The presence of dermoscopic features in the acquired images—namely, vessel morphology and their arrangement, scales and their distribution, lines, circles and structureless areas, as well as their color and arrangement—was evaluated by the authors in consensus before receiving the histopathological report [12].

Dermoscopic features of each NIBP type—namely, LPBP, LSBP, PBP and NSBP—were separately compared to a group that consisted of the other NIBP types using the non-parametric Fisher’s exact test. For example, LPBP was compared to a group of other NIBP that included LSBP, PBP, NSBP and ZBP, while PBP was compared to a group that included LPBP, LSBP, NSBP and ZBP, etc. Given the large number of features examined, unadjusted *p* values were corrected for multiple testing using the Benjamini–Hochberg false discovery rate (FDR) method. All statistical inferences were based on FDR-adjusted *p* values, while unadjusted (raw) *p* values are reported for transparency. *p* values below 0.05 were considered statistically significant. Odds ratios (ORs) and corresponding 95% confidence intervals (CI) were calculated for features demonstrating a positive statistically significant association to quantify strength of association. Statistical analysis was performed using Jamovi (version 2.6; The Jamovi Project (2025), Sydney, Australia) and R (version 4.5.2; Vienna, Austria).

## 3. Results

A total of 78 patients with NIBP were enrolled, including 23 cases of LPBP, 16 cases of PBP, 19 cases of NSBP, 17 cases of LSBP and 3 cases of ZBP.

Frequencies of dermoscopic features of each NIBP type are summarized in Table 1.

**Figure 1:**
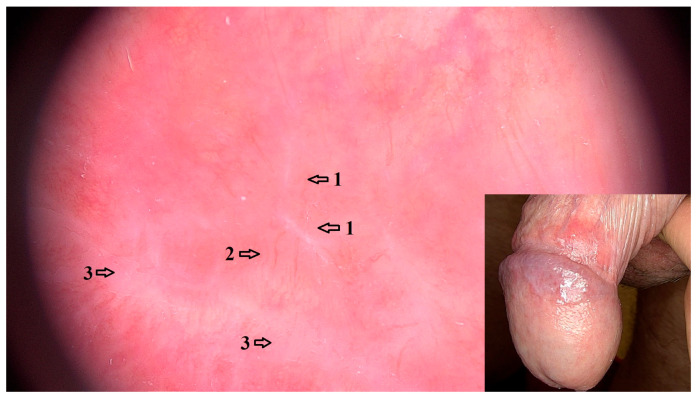
Dermoscopic image (magnification 20×) of LPBP with reticular and angulated white lines (1) that represent WS and peripheral linear vessels (2) with background erythema. Purple structureless areas (3) represent “veil-like” WS. Clinical image of LPBP showing focal erythema of the foreskin and glans, with reticular purple-white WS.

Dermoscopic features that were positively associated with LPBP were reticular white lines (OR = 9.17 95% CI: 2.68–31.40; *p* < 0.001). Purple reticular lines (OR = 19.00 95% CI: 0.94–383.00; *p* = 0.098) and purple focal structureless areas (OR = 2.91 95% CI: 1.07–7.93; *p* = 0.158) were also a common LPBP feature, although the association was not statistically significant. These structures are variations in WS that were dermoscopically observed in 22 (95.65%) cases of LPBP. Linear vessels (OR = 8.20 95% CI: 2.19–30.60; *p* = 0.009) and curved linear vessels (OR = 15.60, 95% CI = 4.49–53.90; *p* < 0.001) at the periphery of WS were also positively associated with LPBP (Figure 1).

**Figure 2:**
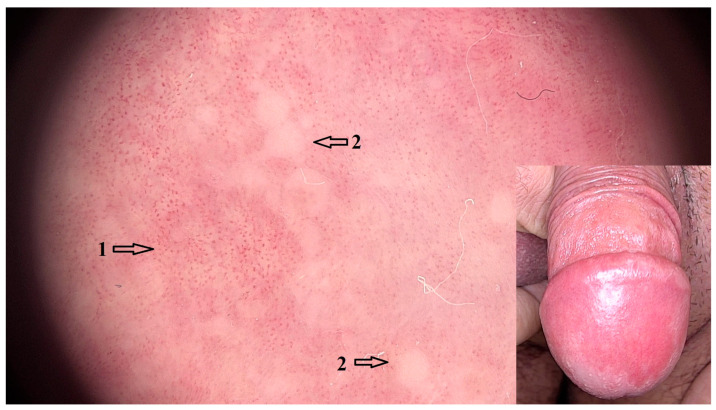
Dermoscopic image (magnification 20×) of PBP with uniformly arranged and clustered dotted vessels (1) and yellow-white globules (2) that represent pustules. Clinical image of PBP showing pronounced erythema of the glans, with pustular lesions at the periphery.

Dermoscopic features that were positively associated with PBP cases were dotted vessels in a uniform (OR = 158.00 95% CI: 8.35–3004.00; *p* < 0.001) and clustered (OR = 5.20 95% CI: 1.58–17.1; *p* = 0.040) arrangement, as well as yellow-white globules (OR = 20.3 95% CI: 2.09–198.00; *p* = 0.031) that corresponded to clinically evident pustules in 2/4 cases (Figure 2). Patchy white scales (OR = 4.64 95% CI: 1.43–14.00; *p* = 0.077) were a common feature, but the association was not statistically significant.

**Figure 3:**
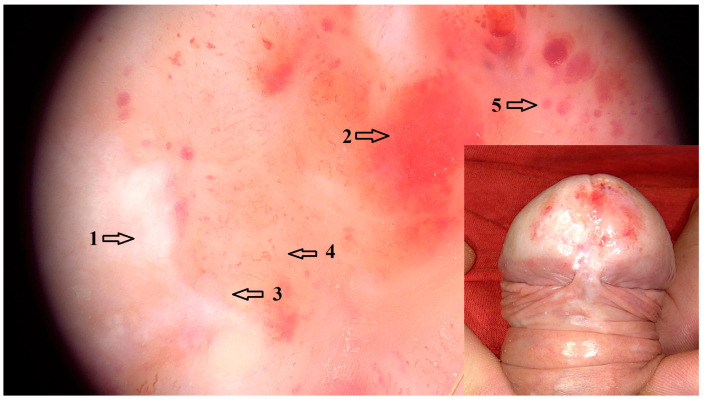
Dermoscopic image (magnification 20×) of LSBP with white (1) and red (2) structureless areas, white lines (3), sparse and unspecifically arranged curved linear vessels (4) and red and purple globules (5). Clinical image of LSBP with erythema and white areas on the glans as well as purpura.

Vascular dermoscopic features that were significantly associated with LSBP included dotted vessels in an unspecific distribution (OR = 5.89 95% CI: 1.76–19.70; *p* = 0.033), linear vessels (OR = 32.70 95% CI: 3.58–299.00; *p* < 0.001) and linear curved vessels (OR = 11.60 95% CI: 2.42–55.10, *p* = 0.009) in an unspecific distribution, as well as decreased vascular density (OR = 1353.00 95% CI: 52.70–34,769.00; *p* < 0.001). Other dermoscopic features that were positively associated with LSBP cases included white structureless areas (OR = 139.00 95% CI: 7.79–2466.00; *p* < 0.001) and white lines (OR = 5.88 95% CI: 1.53–22.60; *p* = 0.038). Particularly white lines in an unspecific arrangement were a common feature of LSBP (OR = 5.00 95% CI: 1.36–18.40; *p* = 0.086), although association with this arrangement was not statistically significant. Red globules (OR = 29.70 95% CI: 1.45–607.00; *p* = 0.049), as well as purple globules (OR = 54.1 95% CI: 2.81–1043.00; *p* < 0.001) were dermoscopic representations of purpura in LSBP (Figure 3). White dots and globules, although a common feature of LSBP, were not statistically significantly associated (OR = 9.08 95% CI: 1.50–54.90; *p* = 0.086).

**Figure 4:**
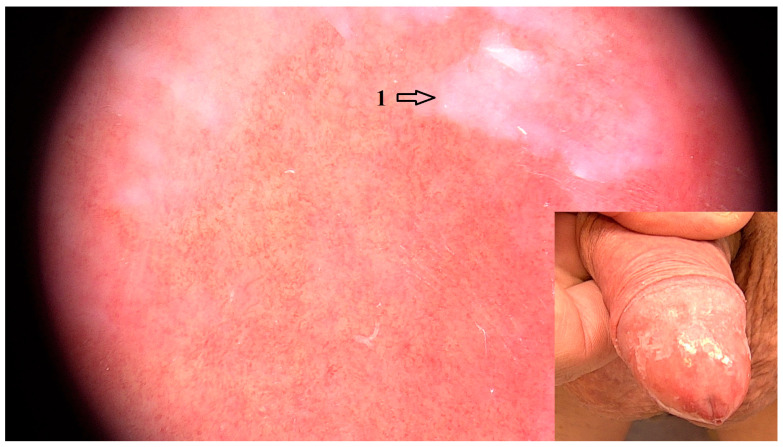
Dermoscopic image (magnification 20×) of NSBP with uniform curved linear vessels, background erythema, white scales and purple-white structureless areas (1) that represent areas of desquamation. Clinical image of NSBP with pronounced erythema on the glans, with areas of desquamation of the epithelium.

Diffuse purple structureless areas were a common feature of NSBP (OR = 4.24 95% CI: 1.40–12.80; *p* = 0.164), but the association was not statistically significant (Figure 4).

**Figure 5:**
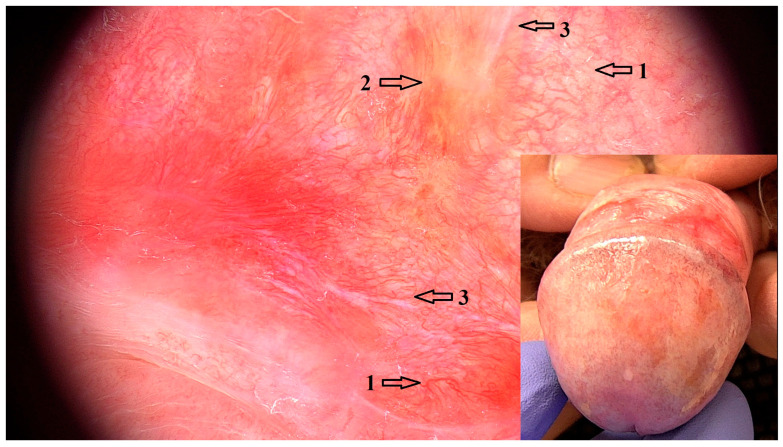
Dermoscopic image (magnification 20×) of ZBP with curved linear vessels with branches (1), orange and yellow structureless areas (2), white lines (3) and red structureless areas. Clinical image of ZBP, with erythema on the glans and foreskin.

Due to the insignificant number of ZBP cases, statistical analysis was not performed. However, commonly observed dermoscopic features included linear vessels with branches in an unspecific distribution (3/3), linear curved vessels in an unspecific distribution (3/3), yellow structureless areas (3/3), orange structureless areas (2/3), purple structureless areas (2/3), white structureless areas (3/3), angulated (1/3) and reticular (2/3) white lines (Figure 5).

## 4. Discussion

Several types of non-infectious balanoposthitis including LPBP, PBP, NSBP and ZBP were enrolled in the study, and each type was associated with certain dermoscopic features. Isolated LPBP was dermoscopically characterized by linear and linear-curved vessels that were arranged at the periphery of the WS. Although WS frequently manifested as white reticular lines, other morphological and color variations were observed including purple reticular lines and purple focal structureless areas that are sometimes referred to as “veil-like” WS in the literature [6]. WS are considered a pathognomonic sign of LP. However, due to the variable morphology, less common variations can be overlooked. The activity of the inflammatory process is a plausible reason for this variability. The dermoscopic patterns of acute and chronic LP had been previously shown to differ in extragenital disease. WS are a characteristic feature of chronic LP lesions and histologically correspond to hypergranulosis. In acute LP, WS can be less obvious, and vascular patterns are the most prominent feature [6].

Certain dermoscopic features of other dermatoses can imitate WS, creating the so-called “pseudo-Wickham striae” [11]. For instance, LSBP presents with white lines similar to those in LPBP. The arrangement of the white lines could help in differentiating both conditions. Reticular arrangement of white lines was common in LPBP, while in LSBP cases, the arrangement was usually unspecific. White structureless areas were also significantly more common in the case of LSBP in comparison to other types of balanoposthitis. Dermoscopy of LSBP has been previously scarcely reported in the literature. Lacarrubba et al. noted that white structures correspond to hyperreflective thickened fibers in the upper dermis during reflectance confocal microscopy (RCM) and, histologically, to dermal fibrosis [8,9]. Red and purple globules were significantly more common in LSBP and clinically corresponded to purpura. Diffuse red structureless areas were present in 38% of LS cases; there was no significant difference in the frequency of erythema in comparison to other NIBP types. Errichetti et al. previously speculated that the presence of reddish areas in LS corresponds to acute dermal inflammation, whereas the presence of white structures indicates a more advanced phase of the disease with dermal fibrosis [8]. Therefore, penile LS could pose a dermoscopic challenge in the absence of white structures, particularly in the early stages of the disease. Histopathologically, LS has been classified into three stages: early pre-slecrotic, sclerotic and atrophic. Attili et al. acknowledged that early histopathological features such as lichenoid and vacuolar interface dermatitis occur in both LS and LP lesions, whereas gross thickening of the basal membrane is a characteristic of LS. Angiogenesis also occurs in the early stages of LS, resulting in dilated blood vessels with thickened walls. In the late stages, vascular structures are absent [13]. In our study, dotted and linear-curved vessels in an unspecific arrangement were the characteristic dermoscopic vascular patterns for LS, distinguishing it from other conditions. We also noted that vessels were less densely distributed, in comparison to other NIBP types. Further studies that compare dermoscopic features of early and advanced LSBP are required. In the absence of white structures, characteristic vascular patterns could be the primary dermoscopic feature of early LSBP.

Uniformly distributed dotted vessels were the dermoscopic hallmark of psoriatic balanoposthitis in our study. The histopathological correlates of this vascular pattern are tortuous, ectatic, elongated capillaries within elongated dermal papillae [9]. White scales in a patchy arrangement were also a common feature of psoriasis, although the association was not statistically significant. These findings correspond to previously published dermoscopic features of PBP, although scales seem to have been reported less frequently in male genital psoriasis than in the current study [5,8,9,10]. Pustules that are seen as white-yellow globules were an additional dermoscopic feature of PBP. In half of these patients, pustules were evident only during dermoscopy and not during clinical inspection. The histopathological basis for the formation of pustular lesions in psoriasis is pyknotic neutrophils in the stratum corneum of the epidermis, known as Munro’s microabscesses, and aggregated neutrophils in the stratum spinosum, commonly referred to as spongiform pustules of Kogoj [14]. To our knowledge there are no studies that report pustules as a dermoscopic feature of genital psoriasis. However, bright, polymorphous cells in the stratum corneum that form Munro’s microabscesses were found to be a characteristic feature during RCM of psoriatic balanitis [15]. Nakanishi et al. demonstrated that dermoscopy significantly improves the detection of histopathologically confirmed pustules in palmoplantar pustulosis in comparison to clinical inspection alone [16]. Therefore, dermoscopy could also enhance visualization of pustules in the case of PBP. Pustular PBP should be differentiated from circinate balanitis, which is characterized by coalescing pustules arranged in a polycyclic pattern. Circinate balanitis is usually associated with human leukocyte antigen (HLA) B27, reactive arthritis, keratoderma blenorrhagicum, uveitis and a recent history of certain infections including Chlamydia trachomatis urethritis [17]. None of these were present in our patients.

It is difficult to draw a definitive clinical distinction between irritant, allergic and NSBP. The space between the foreskin and the glans penis is referred to as the sub-preputial recess. It is an intertriginous site and thus prone to develop irritant balanoposthitis [18]. Ammonia released from urine by bacterial hydrolysis, as well as frequent cleansing with soap, have been implicated as common irritants. Prevalence of balanitis in circumcised men versus uncircumcised is lower by 68%; therefore, the so-called dysfunctional foreskin is considered a contributing causal factor [3,19]. An association between atopic disease and irritant balanoposthitis has also been described in the literature [18]. In the case of allergic balanoposthitis, patch testing should be performed to pinpoint the allergen to which the individual is sensitized [18]. The disease can be referred to as NSBP if it follows a chronic or relapsing course, no specific irritant or allergen has been identified and the histopathological features are also non-specific [3,20]. Circumcision is curative in most cases [3]. In the current study, 19 patients were diagnosed with NSBP. Purple structureless areas were a common dermoscopic feature of NSBP. Diffuse and focal purple structureless areas in NSBP cases clinically corresponded to areas of desquamation (Figure 4). In contrast, focal purple structureless areas in LPBP cases corresponded to “veil-like” WS. None of the dermoscopic features demonstrated a statistically significant association with NSBP. However, linear curved vessels mostly in an unspecific distribution were observed in 17/19 cases. In 8/19 cases, pronounced background erythema was evident. Dotted vessels were also a common finding in 7/19 cases. Our findings are consistent with Errichetti et al., who described blurry linear vessels on a reddish background in four irritant balanitis cases, while sparse dotted vessels were also visualized in two of these cases [8].

ZBP, also referred to as plasma cell balanitis, is a rare inflammatory disease that runs a chronic course in uncircumcised men, aged 40 years and older. Irritation from urine and friction has been implicated as a possible trigger, similarly to NSBP [3]. Clinically ZBP manifests as erythematous macules with a shiny glazed surface that could involve both the glans and the foreskin. Biopsy is often required to rule out other conditions such as LP, LS, psoriasis, fixed drug eruptions and erythroplasia of Queyrat. Dermal plasma cell infiltrate is a hallmark histopathological feature of ZBP. Similar lesions have been described on airways, oral cavity, digestive tract and vulva [7]. Reportedly, ZBP can complicate other conditions, especially LS, and some authors have cast doubt that true ZBP exists [21]. In our study, all the cases of ZBP had no other underlying genital mucocutaneous condition. Linear-curved vessels, with and without branches, as well as yellow and orange structureless areas were the most prominent dermoscopic features. Although the findings are consistent with previous publications, variable terminology had been used to describe the linear vessels, including serpentine, chalice-like and spermatozoa-like. The visualization of vessels is thought to be the consequence of epidermal atrophy and vascular dilation observed on histopathology, while the orange structureless areas are observed due to the extravasation of erythrocytes and the deposition of hemosiderin in the dermis. In contrast, white lines, as well as white and yellow structureless areas that were observed in our study, could correlate with dermal fibrosis, as previously described in ZBP with duration longer than six months. Therefore, older ZBP lesions could pose a diagnostic difficulty due to some dermoscopic similarities to LSBP [22,23].

There were several limitations of the current study. The study included only patients with fair skin. Therefore, the findings cannot be generalized to all populations. We also did not compare the dermoscopic features of lesions on the glans and the foreskin, although some discrepancies are foreseeable due to the differences in anatomical structure of both locations. The study also did not provide any follow-up dermoscopic images of the conditions. The dermoscopic features differ according to the stage and severity of the disease; therefore, sequential dermoscopic monitoring could uncover the prognostic value of dermoscopic features. The study focused only on NIBP. Dermoscopic comparison of NIBP to common infectious and neoplastic penile conditions is essential to interpret dermoscopic findings of penile lesions in a clinical setting. Only the cases in which a biopsy was performed were included in the study. Therefore, the study is focused on NIBP where the authors had doubts about the clinical diagnosis. NIBP cases with a more typical clinical and dermoscopic presentation may have been omitted from this study. These limitations should be addressed in further studies.

## 5. Conclusions

Most common presentation of WS in LPBP are white reticular lines with peripheral linear vessels. However, other morphologies such as focal purple structureless areas are possible. Vascular patterns can help in differentiating WS from pseudo-WS that could be present in LSBP, NSBP and ZBP. The dermoscopic hallmark of PBP are uniformly distributed dotted vessels; however, pustules are another dermoscopic feature that is characteristic of PBP. Background erythema, although common in all types of NIBP, is not always present. There is a significant portion of NIBP cases that lack specific dermoscopic features. Clinical and histopathological correlation is important in these cases for accurate diagnosis. Further studies should correlate the dermoscopic presentation of NIBP to the stage and severity of the disease.

## Figures and Tables

**Table 1:** Comparison of dermoscopic features of non-infectious balanoposthitis (NIBP). Raw and adjusted *p* values are related to the figures written in bold. LPBP—lichen planus balanoposthitis; PBP—psoriatic balanoposthitis; LSBP—lichen sclerosus balanoposthitis; NSBP—non-specific balanoposthitis.

Dermoscopic Criteria, Arrangement	LPBP	PBP	LSBP	NSBP	*p* (Raw)	*p* (Adjusted)
Dotted vessels, uniform	0 (0.00%)	**9 (56.25%)**	0 (0.00%)	0 (0.00%)	<0.001	<0.001
Dotted vessels, clustered	3 (13.04%)	**8 (50.00%)**	3 (17.65%)	4 (21.05%)	0.008	0.040
Dotted vessels, peripheral	**3 (13.04%)**	0 (0.00%)	0 (0.00%)	0 (0.00%)	0.023	0.098 ^3^
Dotted vessels, unspecific	5 (21.74%)	0 (0.00%)	**8 (47.06%)**	3 (15.79%)	0.005	0.033
Linear vessels, peripheral	**9 (39.13%)**	3 (18.75%	1 (5.88%)	0 (0.00%)	0.001	0.009
Linear vessels, unspecific	0 (0.00%)	0 (0.00%)	**6 (35.29%)**	1 (5.26%)	<0.001	<0.001
Linear curved vessels, peripheral	**14 (60.87%)**	2 (12.50%)	1 (5.88%)	2 (10.53%)	<0.001	<0.001
Linear curved vessels, unspecific	5 (21.74%)	5 (31.25%)	**15 (88.24%)**	11 (57.89%)	0.001	0.009
Yellow-white globules	0 (0.00%)	**4 (25.00%)**	0 (0.00%)	1 (5.26%)	0.006	0.031
White scales, patchy	6 (26.09%)	**8 (50.00%)**	3 (17.65%)	2 (10.53%)	0.018	0.077 ^3^
Purple structureless areas, focal	**13 (56.52%)**	1 (6.25%)	7 (41.18%)	7 (36.84%)	0.043	0.158 ^3^
Purple structureless areas, diffuse	6 (26.09%)	4 (25.00%)	8 (47.06%)	**13 (68.42%)**	0.015	0.164 ^3^
Red structureless areas, focal	10 (43.48%)	5 (31.25%)	5 (29.41%)	7 (36.84%)	-	-
Background erythema	9 (39.13%)	9 (56.25%)	6 (35.29%)	8 (42.11%)	-	-
White structureless areas	6 (26.09%)	0 (0.00%)	**17 (100.00%)**	3 (15.79%)	<0.001	<0.001
White dots and globules	1 (4.35%)	1 (6.25%)	**4 (23.53%)**	0 (0.00%)	0.018	0.086 ^3^
White lines	**21 (91.30%)**	1 (6.25%)	**14 (82.35%)**	3 (15.79%)	<0.001 ^1^	<0.001 ^1^
					0.006 ^2^	0.038 ^2^
White lines, reticular	**11 (47.83%)**	0 (0.00%)	4 (23.53%)	0 (0.00%)	<0.001	<0.001
White lines, unspecific	3 (13.04%)	0 (0.00%)	**6 (35.29%)**	3 (15.79%)	0.019	0.086 ^3^
Purple reticular lines	**3 (13.04%)**	0 (0.00%)	0 (0.00%)	0 (0.00%)	0.023	0.098 ^3^
Red globules	0 (0.00%)	0 (0.00%)	**3 (17.65%)**	0 (0.00%)	0.009	0.049
Purple globules	0 (0.00%)	0 (0.00%)	**5 (29.41%)**	0 (0.00%)	<0.001	<0.001

^1^ *p* value for LPBP; ^2^ *p* value for LSBP. ^3^ The FDR adjusted *p* value is not significant.

## Data Availability

Dataset available on request from the authors.

## Abbreviations

The following abbreviations are used in this manuscript:

NIBP	Non-infectious balanoposthitis
LPBP	Lichen planus balanoposthitis
PBP	Psoriatic balanoposthitis
NSBP	Non-specific balanoposthitis
ZBP	Zoon’s balanoposthitis
WS	Wickham striae
PIN	Penile intraepithelial neoplasia
SCC	Squamous cell carcinoma
LP	Lichen planus
LS	Lichen sclerosus
RCM	Reflectance confocal microscopy
HLA	Human leukocyte antigen
